# The Antifungal Activity of Chitosan Nanoparticle-Incorporated Probiotics Against Oral Candidiasis

**DOI:** 10.7759/cureus.70093

**Published:** 2024-09-24

**Authors:** Shree Abiraami N.S., Devika S Pillai, Rajeshkumar Shanmugam

**Affiliations:** 1 Oral Medicine and Radiology, Saveetha Dental College and Hospitals, Saveetha Institute of Medical and Technical Sciences, Saveetha University, Chennai, IND; 2 Nanobiomedicine, Centre for Global Health Research, Saveetha Medical College and Hospital, Saveetha Institute of Medical and Technical Sciences, Saveetha University, Chennai, IND

**Keywords:** chitosan, in vitro antifungal activity, nanoparticles, oral candidiasis, probiotics

## Abstract

Background: Candida species, especially *Candida albicans*, cause oral candidiasis, also known as oral thrush. It affects the elderly, newborns, patients under antibiotics, chemotherapeutic agents, and patients with weaker immune systems. Although successful, traditional antifungal medications are available, they may have negative effects and do not prevent recurrence. Conventional antifungal medications have a restricted range of effectiveness; hence, the need for a novel antifungal agent arises. Chitosan, a natural polymer made from chitin found in crustaceans, kills fungal cells by damaging membranes. Excellent biocompatibility and biodegradability make it a promising medical material. Probiotics, live bacteria, compete with pathogenic microbes for adhesion sites and nutrients, produce antimicrobials such as lactic acid, hydrogen peroxide, and bacteriocins, and modulate the host's immunological response. Adding probiotics to chitosan nanoparticles (CSNPs) boosts their antifungal activity against Candida species and increases their stability and transport to the infection site.

Methods: The antifungal activity was evaluated through in vitro experiments utilizing Candida strains that are significant to oral candidiasis. Different concentrations of CSNPs and formulations loaded with probiotics were examined to assess their effectiveness. The antifungal properties were assessed using microbiological assays, specifically agar diffusion and minimum inhibitory concentration (MIC).

Results: The study demonstrated a notable fungicidal impact of CSNPs combined with probiotics against oral candidiasis. Furthermore, the MIC values were lower for the probiotic-loaded CSNPs than for chitosan alone or probiotics alone.

Conclusion: The combination of CSNPs and probiotics shows potential in effectively combating oral candidiasis by inhibiting fungal growth. This novel method offers a promising strategy for creating therapeutic treatments for oral candidiasis by utilizing the combined benefits of chitosan and probiotics to combat fungal infections effectively.

## Introduction

Oral candidiasis, also known as oral thrush, is a fungal mouth infection caused by the yeast *Candida albicans*. This condition is common, especially in individuals with weakened immune systems, infants, older adults, and those using certain medications [1]. The antifungal properties of chitosan nanoparticles (CSNPs) combined with probiotics represent a groundbreaking research area with significant potential in medicine, agriculture, and food preservation. This combination leverages the unique characteristics of both chitosan and probiotics to enhance their antifungal effects [2]. Chitosan is a natural polymer derived from chitin, found in the exoskeletons of crustaceans like shrimp and crabs. It has several beneficial properties and is renowned for its broad-spectrum antimicrobial qualities, including antifungal activity. Chitosan disrupts fungal cell membranes, leading to cell death. Its biocompatibility and biodegradability make it ideal for pharmaceutical and medical applications [3]. When formulated into nanoparticles, chitosan's surface area increases, improving its interaction with microbial cells and enhancing its efficacy. Nanoparticles also increase the bioavailability of chitosan and probiotics, allowing regulated and sustained release and improving therapeutic efficacy [4].

Probiotics are live bacteria that provide health benefits when administered in adequate amounts. Common probiotic strains include Lactobacillus, Bifidobacterium, and the yeast genus Saccharomyces. Probiotics prevent harmful pathogens' growth by competing for adhesion sites and nutrients [5]. They produce antibacterial compounds such as lactic acid, hydrogen peroxide, and bacteriocins. Additionally, probiotics can boost the host's immune response, helping control disease spread [6]. Combining CSNPs with probiotics can enhance antifungal activity through various mechanisms. Chitosan's antifungal properties, combined with probiotics' competitive exclusion and antimicrobial production, work synergistically to inhibit fungal growth. CSNPs can protect probiotics from environmental stressors, enhancing their stability and delivery to target sites [7]. Nanoparticles can also improve the bioavailability of probiotics and chitosan, allowing for a more controlled release and increased antifungal efficacy [8].

Antifungal drugs, while effective, have several limitations. The development of resistance among fungal strains, particularly *C. albicans*, can reduce the efficacy of treatments and limit available options. These medications can also cause side effects, including organ toxicity and gastrointestinal issues [9]. Additionally, antifungals often interact with other drugs, potentially leading to reduced efficacy or increased toxicity. These limitations highlight the need for careful use and ongoing research to develop new and better antifungal therapies. Research is needed to determine the optimal ratio of CSNPs to probiotics for maximum antifungal effectiveness [10]. Understanding the molecular mechanisms behind this combination is crucial. Ensuring that chitosan-probiotic formulations are safe and nontoxic for human consumption is essential, particularly for medical and food industry applications. Clinical trials are underway to evaluate the efficacy and safety of these formulations in real-world scenarios [11].

Using CSNPs combined with probiotics offers an innovative approach to developing new antifungal treatments, potentially enhancing their effectiveness and safety profiles. Oral candidiasis, a fungal infection caused mostly by Candida species, is a significant concern in dentistry and oral health [12]. The ever-increasing frequency of antifungal resistance and the necessity of finding other therapeutic pathways have prompted research into novel approaches to combat this hardy infection [13]. At the molecular level, the interaction between CSNPs and probiotics in combating fungal infections involves a combination of direct antifungal action from chitosan and the beneficial modulation of the host's microbiota by probiotics. Due to their amino groups, CSNPs, derived from chitin, carry a positive charge [14]. This allows them to interact electrostatically with the negatively charged fungal cell membranes, causing increased membrane permeability, disruption of the cell wall, and leakage of intracellular components, leading to fungal cell death. Additionally, chitosan can penetrate the fungal cell wall and bind to intracellular molecules like DNA, inhibiting replication and other vital functions [15]. Considering this, the combination of CSNPs and probiotics presents a potentially fruitful approach to developing efficient antifungal treatments specifically targeted to oral candidiasis. This study aims to develop a novel therapeutic method by investigating the synergistic potential of mixing CSNPs with probiotics. We hope that by analyzing the antifungal activity of this integrated system, we will be able to contribute to developing novel approaches to managing and preventing oral candidiasis. In doing so, we can address the issues currently associated with standard antifungal treatments.

## Materials and methods

The present study has been approved by the Institutional Human Ethical Committee, Saveetha Dental College and Hospitals, Chennai, India (reference number: IHEC/SDC/OMED CERT-2102/22/TH-096), before data collection. The in vitro investigations were conducted in the Gold Laboratory, Department of Pharmacology, Saveetha Dental College and Hospitals. The CSNPs were formulated in Gold Laboratory, and commercially available probiotics (*Lactobacillus rhamnosus* GG, ATCC 53103) sachets with 10 billion colony-forming units (CFUs) were used in the study.

Formulation of CSNPs

**Figure 1:**
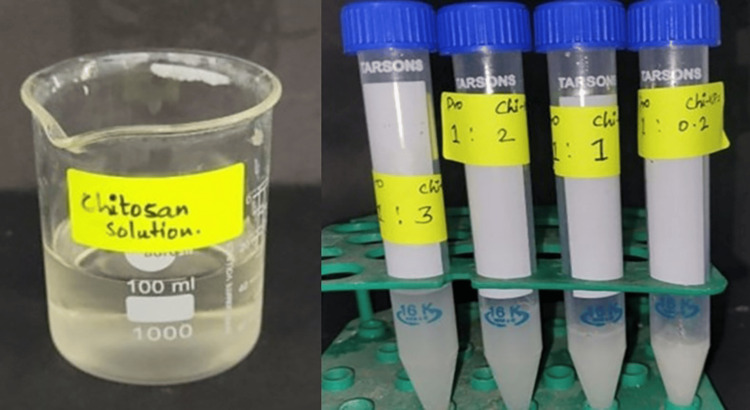
A laboratory setup involving the preparation and analysis of chitosan solutions. On the left side, a clear glass beaker holds a chitosan solution, with the label indicating its contents. The beaker is marked with volume graduations, and the solution is around 100 mL. The liquid appears clear and slightly viscous. On the right side, four clear plastic centrifuge tubes are present, neatly arranged in a green rack. Each tube has a label, indicating different ratios or concentrations of chitosan, "1: 2 Chi," "1: 1 Chi," "1: 3 Chi," and "0.2 Chi." The tubes contain a cloudy white substance, representing varying concentrations of chitosan preparations for experimental comparison

The formulation of CSNPs involves preparing a chitosan solution by dissolving chitosan powder in an acidic medium, typically acetic acid, to obtain a clear solution. The concentration of chitosan is carefully measured to achieve the desired consistency. The solution is then stirred continuously to ensure complete dissolution. Next, a crosslinking agent, such as sodium tripolyphosphate (TPP), is gradually added to the chitosan solution under constant stirring. The ratio of chitosan to TPP is varied to control the size and properties of the nanoparticles. This mixture is then subjected to high-speed stirring or ultrasonication to promote the formation of nanoparticles. The resulting suspension is centrifuged to separate the nanoparticles, which are then washed with distilled water to remove residual agents. Finally, the nanoparticles are collected, often by freeze-drying and stored for further analysis or use in biomedical applications. The concentration of probiotics remained constant in one sachet, while different concentrations of CSNPs were added at ratios of 1:0.2, 1:1, 1:2, and 1:3 (Figure 1).

Assessment of antifungal activity

**Figure 2:**
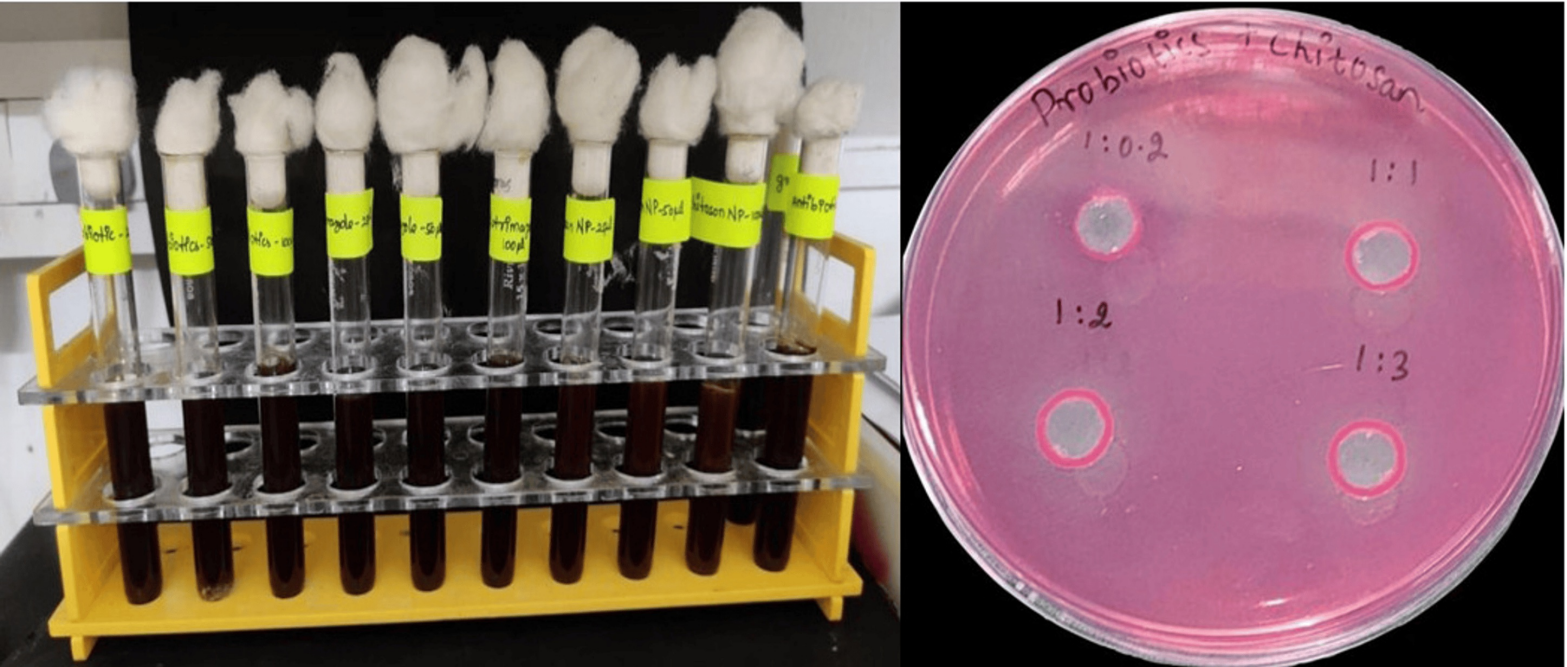
Laboratory experiments involving chitosan, proteins, nanoparticles, and probiotics. On the left, 10 glass test tubes are arranged in a yellow plastic rack, each filled with a dark brown liquid and sealed with cotton plugs. The test tubes have labels indicating different contents or experimental conditions, such as "protein-S," "chitosan-1," "NP-sol," and variations thereof, suggesting a study of interactions between these components. On the right, a Petri dish with a pink agar medium is labeled "probiotics+chitosan." The dish features four circular spots marked with different ratios: "1: 0.2," "1: 1," "1: 2," and "1: 3," representing varying concentrations of the chitosan-probiotic mixtures. The clear zones around these spots on the pink medium indicate microbial growth inhibition, reflecting the effectiveness of the different chitosan formulations being tested

The agar well diffusion method and the time-kill assay determined the efficacy of CSNPs containing probiotics at different concentrations. In the agar well diffusion method, nutrient agar was prepared, sterilized, and poured into Petri dishes to solidify. A standardized fungal suspension was spread evenly across the surface of the solidified nutrient agar using a sterile swab to ensure uniform fungal growth. Wells, typically 6-8 mm in diameter, were created in the agar using a sterile cork borer or similar tool, ensuring they were evenly spaced and sufficiently separated to prevent overlapping inhibition zones. Different concentrations of CSNPs containing probiotics were prepared, and a measured volume (e.g., 50-100 µL) of each solution was carefully pipetted into each well. The antifungal activity was assessed by measuring the inhibition zones around the wells, indicating the nanoparticles' effectiveness against the fungal strain (Figure 2).

Time-kill curve assay

The time-kill curve assay began with the preparation of reinforced broth agar, which was sterilized and then dispensed in 6 mL aliquots into five test tubes. A suspension of Candida, standardized to a concentration of 5 x 10^5^ CFU/mL, was added to each test tube. The antifungal treatment, comprising CSNPs containing probiotics at various concentrations, was then introduced to the test tubes. Samples were collected at different time intervals (e.g., 0, 2, 4, 8, 12, and 24 hours) to monitor the effectiveness of the treatment over time. Each sample was plated on nutrient agar and incubated to allow colony formation. The number of viable colonies was counted at each time point to generate a time-kill curve, illustrating the reduction in Candida CFU/mL throughout the treatment, thereby providing a dynamic view of the antifungal efficacy of the CSNP formulation.

Cytotoxicity evaluation

To evaluate the potential cytotoxicity of the formulated CSNP solution infused with probiotics, a solution was prepared by dissolving 2 g of iodine-free salt in 200 mL of distilled water. Six-well enzyme-linked immunosorbent assay (ELISA) plates were filled with 10-12 mL of this saline solution in each well. Nauplii were then introduced into each well at varying volumes of 5, 10, 20, 40, and 80 µL, respectively, while the sixth well was maintained as a control with no treatment. Different concentrations of the CSNPs containing probiotics were added to the wells corresponding to the volumes of nauplii. Following a 24-hour incubation period, the wells were observed for any signs of cytotoxic effects, such as nauplii mortality or morphological changes. The observations were documented meticulously to assess the safety of the nanoparticle formulations. This cytotoxicity evaluation provided crucial insights into the biocompatibility of CSNPs combined with probiotics, supporting their potential application in treating oral candidiasis by demonstrating minimal adverse effects on cell viability.

Statistical analysis

The data generated were subjected to statistical analysis through the IBM SPSS Statistics for Windows, version 22.0 (released 2013; IBM Corp., Armonk, NY). The Kruskal-Wallis test was performed with a significance level set at p ≤ 0.05.

## Results

**Figure 3:**
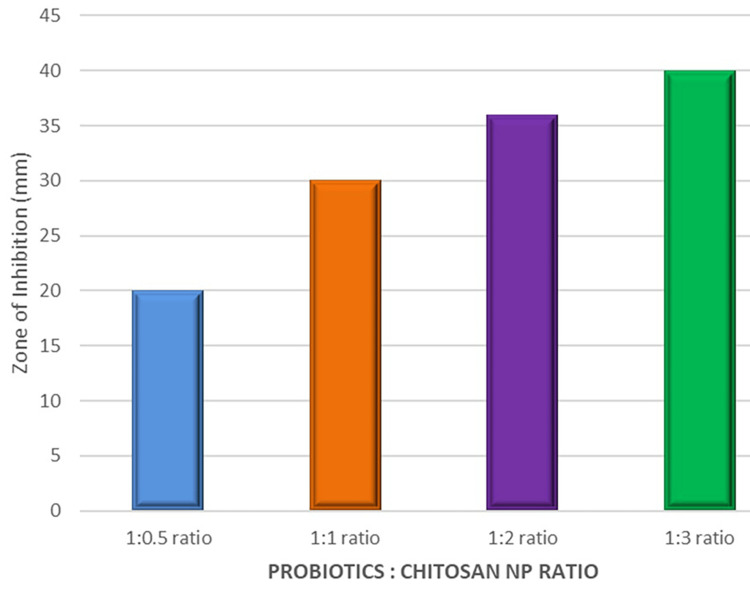
The relationship between the ratio of probiotics to chitosan and the corresponding antimicrobial efficacy, as measured by the zone of inhibition in millimeters. As the ratio of chitosan increases relative to probiotics, there is a notable increase in the zone of inhibition, suggesting enhanced antimicrobial activity. Specifically, the zone of inhibition grows from 20 mm at a 1:0.5 ratio to 40 mm at a 1:3 ratio, indicating that a higher proportion of chitosan significantly boosts the antimicrobial effect. This trend underscores the potential of chitosan as a powerful antimicrobial agent when used in conjunction with probiotics NP: nanoparticle

The agar well diffusion method evaluated the antifungal activity of CSNPs mixed with varying amounts of probiotics. The CSNPs derived from yeast were combined with probiotics in different ratios to observe their effectiveness against fungal infections. The results were as follows: the 1:0.2 ratio displayed an inhibition zone of 20 mL, the 1:1 ratio showed a more significant inhibition zone of 30 mL, the 1:2 ratio further increased the inhibition zone to 36 mL, and the 1:3 ratio achieved the largest inhibition zone of 40 mL. These results indicated that the antifungal activity improved with increasing amounts of probiotics, chitosan, and clotrimazole of probiotics mixed with the CSNPs, with the 1:3 ratio showing the most potent effect (Figure 3).

**Figure 4:**
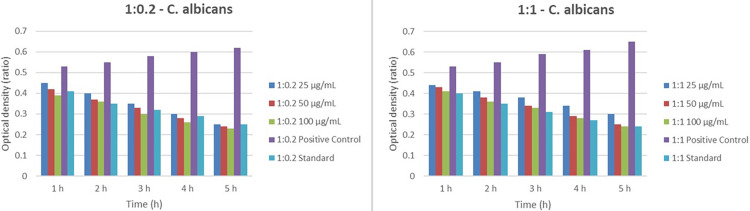
Comparison of the OD of C. albicans at two different ratios of treatment (1:0.2 and 1:1) over a five-hour period, with varying concentrations of the treatment (25, 50, and 100 µg/mL), alongside a positive control and a standard. In both graphs, the optical density decreases over time for all treatment concentrations, indicating a reduction in C. albicans growth. The positive control consistently shows the highest optical density, while the standard demonstrates the lowest. The 1:0.2 ratio shows a more pronounced decrease in optical density across all time points compared to the 1:1 ratio, suggesting that the 1:0.2 ratio is more effective in reducing C. albicans growth. Additionally, higher concentrations of the treatment (100 µg/mL) result in lower optical density, further indicating greater efficacy in inhibiting C. albicans growth. Overall, the data illustrate that both treatment ratios and higher concentrations are effective in reducing C. albicans growth, with the 1:0.2 ratio showing superior performance OD: optical density

In the 1:2 and 1:3 ratios, the optical density decreases progressively across the five-hour period, indicating a reduction in* C. albicans* growth. The positive control consistently shows the highest optical density, while the standard exhibits the lowest values. Notably, the 1:3 ratio demonstrates a more significant reduction in optical density across all time points than the 1:2 ratio, suggesting a greater effectiveness in inhibiting *C. albicans* growth. Higher treatment concentrations (100 µg/mL) also lead to lower optical density, further indicating enhanced efficacy at these concentrations. The data suggest that the 1:2 and 1:3 treatment ratios effectively reduce *C. albicans* growth, with the 1:3 ratio showing superior performance (Figure 4).

**Figure 5:**
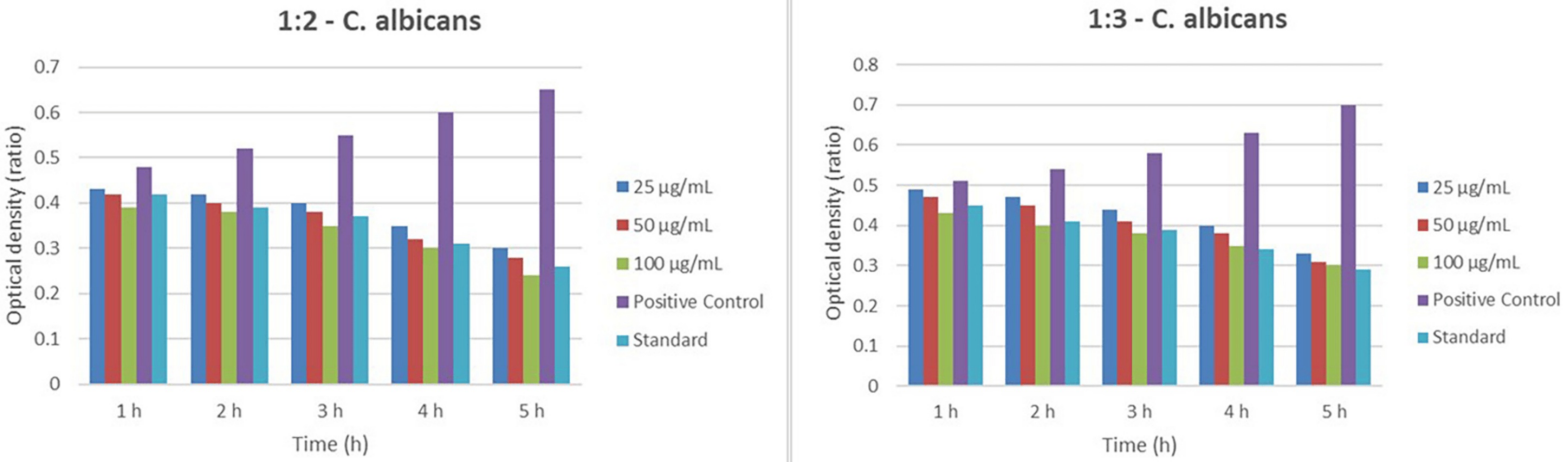
The OD of C. albicans over a five-hour period, comparing the effectiveness of two treatment ratios, 1:2 and 1:3, with varying concentrations of the treatment (25, 50, and 100 µg/mL). Each treatment's impact is measured against a positive control and a standard OD: optical density

The time-kill assay provided additional insights into the bactericidal activity of the different ratios over time. Among the tested ratios, the 1:3 ratio again outperformed the others, demonstrating superior bactericidal activity throughout the duration of the assay. This dynamic assessment reinforced the findings from the agar well diffusion method, highlighting the effectiveness of the higher probiotic concentration in the CSNP formulation (Figure 5).

**Figure 6:**
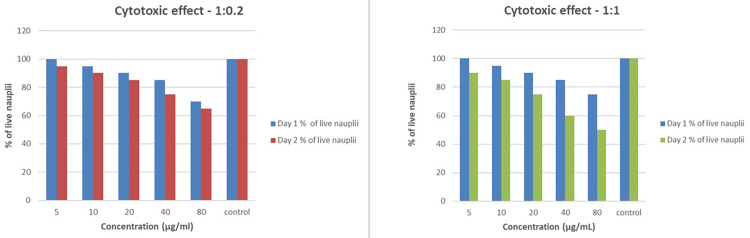
Comparison of the cytotoxic effects of two concentration ratios (1:0.2 and 1:1) on live nauplii over two days. Both graphs show a concentration-dependent decrease in the percentage of live nauplii, with higher concentrations (40 and 80 µg/mL) leading to a more significant reduction in viability. The control groups maintained nearly 100% live nauplii, while the 1:1 ratio exhibited a slightly stronger cytotoxic effect than the 1:0.2 ratio

A cytotoxicity test was conducted to ensure the safety of the formulated solutions. The test aimed to determine whether the solutions could potentially harm cell viability. The evaluation involved dissolving 2 g of iodine-free salt in 200 mL of distilled water and filling six-well ELISA plates with 10-12 mL of this saline solution. Nauplii were introduced into each well at volumes of 5, 10, 20, 40, and 80 µL, respectively, with the sixth well serving as a control. Various concentrations of the CSNPs infused with probiotics were then added to the wells based on the volumes of nauplii. After a 24-hour incubation period, the results showed that all ratios exhibited very minimal cytotoxic effects. Notably, the 1:0.2 ratio displayed the least cytotoxicity, suggesting a favorable safety profile (Figure 6).

**Figure 7:**
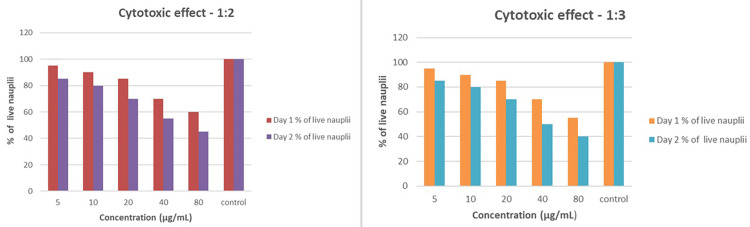
The cytotoxic effects of two concentration ratios, 1:2 and 1:3, on live nauplii over two days. Both ratios show a concentration-dependent decrease in the percentage of live nauplii, with higher concentrations (40 and 80 µg/mL) leading to a significant reduction in viability. The 1:3 ratio exhibits a more pronounced cytotoxic effect than the 1:2 ratio, particularly at higher concentrations. In both cases, the control groups maintained nearly 100% viability, underscoring the impact of increasing concentration on nauplii survival

**Figure 8:**
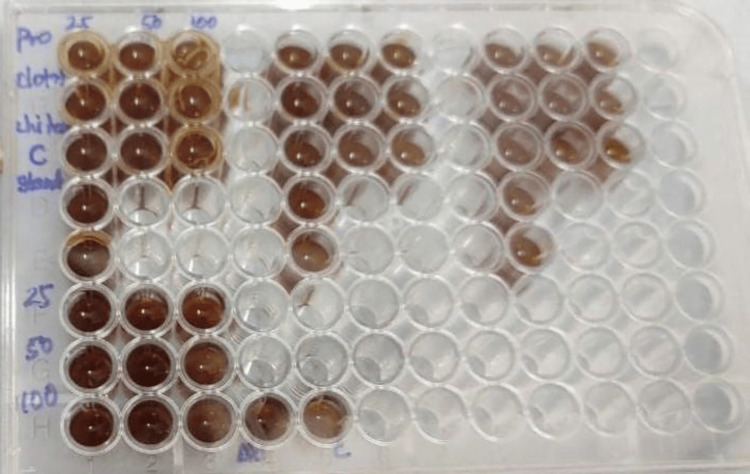
A 96-well microplate used in a time-kill assay to determine the MIC of probiotics, chitosan, and clotrimazole MIC: minimum inhibitory concentration

This low cytotoxicity across different concentrations indicates that combining CSNPs with probiotics has a low risk of harming cell viability. These findings suggest the combination has a favorable safety profile for potential therapeutic applications. Given the demonstrated antifungal activity and low cytotoxicity, this combination appears promising for further exploration in treating oral candidiasis. The intriguing antifungal activity, particularly at the 1:3 ratio, coupled with the minimal cytotoxic effects, points to positive outcomes that could result from using this combination in clinical applications (Figure 7). The minimum inhibitory concentration of probiotics, chitosan, and clotrimazole was tested using a time-kill assay (Figure 8).

## Discussion

Oral candidiasis, commonly caused by the fungal pathogen *C. albicans*, is particularly problematic in immunocompromised individuals, necessitating the development of effective antifungal treatments. CSNPs possess intrinsic antimicrobial properties primarily due to their polycationic nature. This characteristic facilitates the interaction with negatively charged microbial cell membranes, resulting in increased permeability and eventual cell lysis [16]. Furthermore, chitosan's ability to chelate metals disrupts microbial enzyme systems and interferes with nutrient uptake. When probiotics, such as Lactobacillus species, are incorporated, the antifungal efficacy is enhanced through multiple mechanisms [17]. Probiotics produce organic acids, hydrogen peroxide, and bacteriocins that inhibit Candida growth. The combined effect of CSNPs and probiotics leads to a multifaceted attack on the fungal cells, disrupting cell wall integrity and metabolic processes more effectively than either agent alone [18].

CSNPs are advantageous due to their biocompatibility and biodegradability, making them suitable for oral applications with minimal side effects [19]. Their nanoscale size enhances their penetration and retention in the oral mucosa, providing prolonged antifungal activity. Additionally, CSNPs can be functionalized to improve their targeting capabilities and control the release of active agents, thereby increasing their therapeutic efficacy. The role of probiotics in oral health is well documented. They help maintain a balanced oral microbiome by competing with pathogens for adhesion sites, producing antimicrobial substances, and modulating the host immune response. Probiotics such as *L. rhamnosus* and Bifidobacterium spp. have been shown to reduce the colonization of Candida species in the oral cavity [20]. The synergistic effect of probiotics and CSNPs targets the fungal cells and promotes the restoration of the natural microbial balance, potentially reducing the recurrence of oral candidiasis. Integrating CSNPs and probiotics represents a promising approach to treating oral candidiasis. Clinical studies are required to assess this combination therapy's safety, efficacy, and optimal dosage. Initial in vitro studies have demonstrated significant antifungal activity, but clinical trials are essential for translating these findings into practical applications [21]. Factors such as the severity of infection, patient compliance, and underlying health conditions must be considered when designing treatment protocols.

CSNPs combined with probiotics have demonstrated significant antifungal effects against oral candidiasis. The findings from the agar well diffusion method and the time-kill assay support this. These findings add to the existing literature that supports the potential of innovative strategies in combating oral infections, specifically those caused by Candida species. Based on the results obtained from the agar well diffusion method, it was observed that the effectiveness of the antifungal treatment increased in a manner that was directly proportional to the dosage [22]. The areas of inhibition zones expanded in direct proportion to the concentration of CSNPs used. These findings align with previous research showcasing chitosan's intrinsic antimicrobial characteristics. There could be a potential relationship between the concentration of nanoparticles and the suppression of Candida growth, as evidenced by the increased inhibition zones. The antifungal activity of CSNPs incorporated with probiotics offers a significant advancement in treating oral candidiasis. This innovative approach combines the antimicrobial properties of CSNPs with the probiotic benefits of maintaining a balanced oral microbiome [14-16]. The antifungal efficacy is additionally substantiated by the time-kill assay, which illustrates that a ratio of 1:3 exhibits superior bactericidal activity over time. Given its consistent effectiveness, it is evident that the developed solution can maintain its antifungal properties over a prolonged duration. This is crucial in advancing efficacious therapies for oral candidiasis conditions [23]. While promising, further research is necessary to fully understand the therapeutic potential and ensure the safe and effective application of this novel treatment in clinical settings.

The minimal cytotoxic effects observed in the cytotoxicity test, especially in the 1:0.2 ratio, provide a reason for optimism. Based on this, the combination of CSNPs and probiotics is well accepted and has a negligible impact on cell viability, even at higher concentrations. However, it is crucial to recognize that further research, potentially involving experiments on living organisms, is necessary to confirm the safety and efficacy of these formulations. Although these findings indicate the potential of combining CSNPs with probiotics, it is crucial to consider the intricate nature of the oral microbiome and the multifactorial aspects of oral candidiasis. Subsequent studies should examine the underlying mechanisms behind the observed antifungal effects, evaluate the long-term safety, and consider potential synergies with existing treatments.

Although the results are proven with in vitro analysis, in vivo studies with large groups of patients and long-term follow-up to analyze the resolution of the candidal growth are required to establish the effectiveness of probiotics with chitosan in managing candidiasis.

## Conclusions

In summary, this study's results emphasize the favorable antifungal properties and minimal harm to cells of CSNPs that were combined with probiotics to address oral candidiasis. Consequently, this sets the stage for the development of inventive and effective treatments in the realm of oral healthcare. The study establishes a basis for future research in this area, offering valuable insights into the investigation of alternative treatments for oral candidiasis and contributing to the ongoing exploration of these treatments.

Future research should focus on optimizing the formulation and delivery methods of CSNP-incorporated probiotics. It is crucial to investigate the long-term effects of this therapy on the oral microbiome and overall health. Additionally, exploring the potential of combining CSNPs with other natural antifungal agents could further enhance therapeutic outcomes. Understanding the molecular interactions between CSNPs, probiotics, and Candida species will provide deeper insights into the underlying mechanisms and pave the way for developing more effective antifungal treatments.
